# Effectiveness of hypnotherapy for paediatric abdominal pain in primary care: a randomised controlled trial

**DOI:** 10.3399/BJGP.2025.0459

**Published:** 2026-05-19

**Authors:** Ilse N Ganzevoort, Marjolein Y Berger, Michiel R de Boer, Marc A Benninga, Arine M Vlieger, Gea A Holtman

**Affiliations:** 1 Department of General Practice and Elderly Care Medicine, University Medical Center Groningen, University of Groningen, Groningen, the Netherlands; 2 Department of Pediatric Gastroenterology, Emma Children’s Hospital, Amsterdam UMC, University of Amsterdam, Amsterdam, the Netherlands; 3 Department of Pediatrics, St. Antonius Hospital, Nieuwegein, the Netherlands

**Keywords:** child, functional abdominal pain, hypnosis, irritable bowel syndrome, primary health care

## Abstract

**Background:**

Functional abdominal pain (FAP) and irritable bowel syndrome (IBS) are associated with anxiety, depression, school absenteeism, and reduced quality of life in children. In secondary care, hypnotherapy is often available.

**Aim:**

To determine the effectiveness of home-based guided hypnotherapy for children with FAP or IBS in primary care.

**Design and setting:**

This was a pragmatic randomised controlled trial in Dutch primary care with a follow-up of 12 months.

**Method:**

Children aged 7–17 years with FAP or IBS diagnosed by a GP were included. The intervention group received home-based guided hypnotherapy via a website for 3 months in addition to care as usual. The control group received care as usual only. The primary outcome was adequate relief of abdominal pain and/or discomfort at 12 months. Adjusted odds ratios (aORs) and 90% confidence intervals (90% CIs) are reported.

**Results:**

In total, 152 children were included. No significant differences were observed in adequate relief at 12 months, 79.5% in the intervention group (58/73 children) versus 74.3% in the control group (52/70 children); aOR 1.47 (90% CI = 0.74 to 2.93), but more children in the intervention group had adequate relief compared with the control group at 3 months (aOR 2.74, 90% CI = 1.22 to 6.13) and 6 months (aOR 4.26, 90% CI = 1.81 to 10.04). The intervention group also showed reductions in abdominal pain severity, intensity, frequency, and threat, and higher proportions of fast sleep onset.

**Conclusion:**

Although adequate relief after 12 months is high in both groups, adding hypnotherapy to care as usual may help achieve earlier relief and reduce pain.

## How this fits in

Hypnotherapy has been found effective in reducing abdominal pain in children with functional abdominal pain (FAP) and irritable bowel syndrome (IBS) in secondary care, but has not been evaluated in primary care to the authors’ knowledge. As most children with FAP or IBS present to their GP with milder symptoms, and hypnotherapy is a low-cost and accessible treatment, evidence from the primary care setting is needed. This study found no difference in adequate relief at 12 months, with high relief rates in both groups. Home-based guided hypnotherapy added to care as usual could potentially lead to earlier pain reduction and sleep improvements compared with care as usual alone, and may therefore be considered by GPs as an additional treatment option for children with FAP or IBS.

## Introduction

Functional abdominal pain (FAP) and irritable bowel syndrome (IBS) are common disorders in children and are associated with higher anxiety and depression scores, school absenteeism, and lower quality of life.^1–4^ In the Netherlands, all children first present to their GP, where management consists of education, reassurance, and appropriate follow-up, focusing on pain management rather than resolution.^5^ However, the current approach seems insufficient: 50% of children still report abdominal pain having an impact on daily activities after 1 year^6^ and nearly 30% after 5 years.^7^ Around 13% are eventually referred to secondary or tertiary care because of severely impaired functioning.^2,8^

Given the strong association between disorders of gut–brain interaction and psychological complaints, children with FAP or IBS may benefit from psychosocial interventions. Hypnotherapy is effective in secondary care for reducing abdominal pain in children and is recommended by the European and North American Societies for Pediatric Gastroenterology, Hepatology and Nutrition.^9–11^ It induces an altered state of consciousness involving focused attention, enabling therapeutic suggestions to alter physiology, sensation, emotion, or thought.^12^ Home-based guided hypnotherapy, which involves listening to exercises at home, reduces cost and time for clinicians, parents, and children compared with face-to-face hypnotherapy. It has been proven non-inferior, showing adequate pain relief in children at 12 months’ follow-up (75% and 87%, respectively).^13^ Benefits are greatest in children with shorter symptom duration, consistent with patients seen in primary care.^13,14^

Home-based guided hypnotherapy could offer GPs a low cost and easy-to-use management option, potentially reducing healthcare resource use (details available from the authors on request). However, its effectiveness in primary care remains unestablished, to the authors’ knowledge, which is important as treatment response may differ from secondary care.^15^ This study aimed to evaluate the effectiveness of home-based guided hypnotherapy in children with FAP or IBS in primary care.

## Method

### Design and setting

This study was a pragmatic, parallel-arm, randomised controlled trial (RCT). Children were recruited by GPs in the northern part of the Netherlands from October 2020 until November 2023, and the last date of follow-up was 6 December 2024. As a result of a slow inclusion rate, additional recruitment was implemented using adverts and (social) media from October 2022. Details of the design, recruitment strategies, outcomes, and informed consent procedure have been reported previously.^16^ The Consolidated Standards of Reporting Trials (CONSORT) guideline and the extension for pragmatic trials was followed.^17,18^ The Medical Ethical Review Board of the University Medical Center Groningen approved the study (METc-number: 2020/237) and the amendment for extending recruitment. The trial was registered at ClinicalTrials.gov (NCT05636358). Informed consent was given by both parents (age <12 years), both parents and the child (age 12–15 years), or the child only (age 16–17 years).

### Participants

Children aged 7–17 years were included if they had chronic gastrointestinal symptoms (such as, recurrent abdominal pain for ≥2 months or ≥2 episodes in the past 2 months) and GP-diagnosed FAP or IBS. The Dutch guideline for GPs defines this as abdominal pain that is not caused by an underlying somatic disease based on medical history and physical examination.^5^ Exclusion criteria included any concomitant organic gastrointestinal disease, abdominal symptoms treated by a paediatrician, intellectual disability, psychotic disorders, a history of hypnotherapy in the past year, or poor comprehension of the Dutch language. For all participants, including those recruited via (social) media, the inclusion and exclusion criteria were confirmed by their own GP. After receiving informed consent, children and/or parents completed baseline questionnaires, including the Rome IV criteria,^19^ before randomisation.

### Randomisation and blinding

Children were randomly allocated to intervention or control at a ratio of 1:1 using a computer-generated randomisation list. A methodologist who had no role in recruitment or trial procedures (the third author) performed randomisation and informed researchers of the result. Allocation was stratified by age (<12 years or ≥12 years), using varying block sizes of four, six, and eight. Participants, GPs, and researchers were not blinded to treatment allocation for data collection. GPs and children in the control group were informed about what hypnotherapy entails and why it might help, but not how to perform hypnotherapy. Researchers were blinded to treatment group allocation during analysis.

### Interventions

#### Control group: care as usual

The control group received care as usual by their GP according to the Dutch guideline for abdominal pain in children comprising education, reassurance, and symptom monitoring.^5^ An English summary of this guideline is available in the protocol paper.^16^ It was assumed that all GPs work according to the Dutch guideline, while allowing for clinically appropriate deviations as part of usual care. Control participants were asked not to engage in hypnotherapy. After follow-up was complete, participants were granted access to the online hypnotherapy intervention, if desired.

#### Intervention group: care as usual plus home-based guided hypnotherapy

Children in the intervention group received home-based guided hypnotherapy in addition to usual GP care. After instructions via video call, the intervention was accessed via a website that comprised five audio-exercises: one breathing/relaxation exercise plus four visualisation exercises, adapted for age (<12 years and ≥12 years); the exercise duration remained constant. The protocol was based on the Manchester^20^ and van Tilburg protocols,^21^ and previously recorded for a hospital-based study.^13^ Exercises aimed to promote relaxation, improve control over abdominal pain and gut function, and strengthen ego. Children were instructed to listen ≥5 days/week, for 15–20 min/day, for 3 months.

### Outcomes

The primary outcome was the proportion of children with adequate relief of abdominal symptoms at 12 months’ follow-up, with the question: ‘Overall, did you have relief of abdominal pain and/or discomfort in the past week, compared with baseline?’ (yes/no). Eight children with functional abdominal pain or irritable bowel syndrome were asked about the outcome ‘adequate relief’ and their recommendations informed the authors’ choice of this term as the study effect outcome. Secondary outcomes were adequate symptom relief at 3 and 6 months’ follow-up; pain severity, intensity, and frequency; school absence; health-related quality of life scores; pain beliefs; anxiety and depression scores; somatisation; and sleep disturbances (Box 1). Outcomes were measured at baseline, 3, 6, and 12 months’ follow-up.^16^ Adverse events were recorded through spontaneous reporting; no specific questions were asked.

**Box 1.** Details of outcomes

Outcome	Instrument	Score calculation and interpretation
**Pain severity**	Numerical rating scale-11 (NRS-11)	Severity of pain and/or discomfort in the past week is rated on an 11-point NRS scale from 0 (no pain) to 10 (worst pain). Higher scores indicate higher pain severity
**Pain intensity and frequency**	Abdominal pain diary of 7 consecutive days	Pain intensity score: an affective facial pain scale consists of nine faces, ranging from no pain at all (score 0) to the most severe pain (score 3). The pain intensity score is the sum score of 7 days (range 0–21) Pain frequency score: pain duration is measured in categories, ranging from no pain (score 0), 1–30 min (score 1), 31–120 min (score 2), to >120 min (score 3). The pain frequency score is the sum score of 7 days (range 0–21)
**Health-related Quality of Life (HR-QoL**)	KIDSCREEN-52 questionnaire	Fifty-two items are covered in 10 HR-QoL dimensions: physical wellbeing, psychological wellbeing, moods and emotions, self-perception, autonomy, relations with parents and home life, social support and peers, school environment, social acceptance (bullying), and financial resources. Each item is rated on a five-point Likert scale. Rasch scores are computed for each dimension, and are then converted to T-value norms with a mean of 50 (standard deviation of 10). Higher T-values indicate a better HR-QoL and wellbeing
**Pain beliefs**	Pain Beliefs Questionnaire (PBQ)	A total of 32 items consists of a pain belief statement that range from not true at all (score 0) to very true (score 4). Three subscales are the sum of their items: pain threat (20 items), problem-focused coping efficacy (six items), and emotion-focused coping efficacy (six items). A higher score on the pain threat scale indicates stronger belief that their abdominal pain is a threat (range: 0–4). Higher scores on both coping subscales indicate stronger beliefs in their ability to cope with pain (range: 0–4)
**Anxiety and depression scores**	Revised Anxiety and Depression Scale-short version (RCADS-25)	This questionnaire comprises 20 items of anxiety and five items of depression disorders. Items are rated on a four-point scale from 0 (never) to 3 (always). A total score for anxiety and depression is the sum of their corresponding items. Higher scores indicate more symptoms of anxiety (range: 0–60) or depression (range: 0–15)
**Somatisation**	Children’s Somatisation Inventory (CSI)	Physical symptoms in the previous 2 weeks are rated on 35 items using a five-point Likert scale ranging from no problems (score 0) to a lot (score 4). A total score is the sum of all items, with higher scores indicating more somatic complaints (range: 0–140)
**Sleep disturbances**	Dutch Sleep Self-Report questionnaire	Three items are questioned: ‘Do you fall asleep in about 20 minutes?’ (score reversed), ‘Do you wake up at night when your parents think you are asleep?’, and ‘Do you feel sleepy during the day?’ The frequency in the past week is rated as rarely (0–1 times), sometimes (2–4 times), and usually (5–7 times). A higher frequency indicates more sleep disturbances

### Statistical analysis

#### Sample size

The initial sample size calculation indicated that 200 participants were needed to detect a 20% difference in treatment success between groups (55% in the control group^6^ versus 75% in the intervention group^13^) with 80% power at a 5% significance level, accounting for 10% loss to follow-up.^16^ As a result of slow recruitment during the trial, the authors accepted an adjustment of significance level from 5% to 10%, which required at least 148 children.

#### Data analysis

Participants were primarily analysed on an intention-to-treat (ITT) basis. The ITT population consisted of all patients who gave informed consent and were randomly allocated to one of the two treatment groups, regardless of treatment adherence. Secondary per-protocol (PP) analyses were performed among children who did not perform hypnotherapy in the control group, and children who started at least four out of five exercises in the intervention group, based on usage data from the website.

The primary outcome was analysed by logistic regression analysis. The secondary outcomes were analysed using multilevel mixed-effects regression models to investigate longitudinal differences between groups. Analysis models included a random intercept at the individual level and estimated effects for the treatment variable, dummy variables for time (3, 6, and 12 months), and the interaction between treatment and time and relevant covariates: age (continuous), treatment expectation, baseline abdominal pain severity score, and the outcome’s corresponding baseline score.

All statistical tests were two-sided using a significance level of 0.1. Adjusted odds ratios (aORs) and adjusted beta-coefficients (aBetas) with 90% confidence intervals (90% CIs) were reported. A post hoc sensitivity analysis was performed by age (restricted to children <12 years), and post hoc subgroup analyses were performed by gender and recruitment strategy (GP recruited versus media recruited).

If >5% was missing, the primary outcome was imputed and observed values were explored to consider items missing at random. Multiple imputation by chained equations using logistic regression with augmentation was used to impute missing values for the primary outcome and covariates. Twenty imputed datasets were created using the following predictors: age, gender, group allocation, diagnosis, symptom duration, baseline expectation of child, baseline pain severity score, quality-adjusted life years, costs, and adequate relief at 3 and 6 months. Results were pooled using Rubin’s rules.^22^ Mean imputation was applied to abdominal pain day diary scores when ≥4 days were completed. Missing data were not imputed in the longitudinal analysis, because this is generally considered unnecessary for mixed models.^23^

Analyses were performed using Stata Statistical Software (Release 18).

## Results

**Figure 1.**
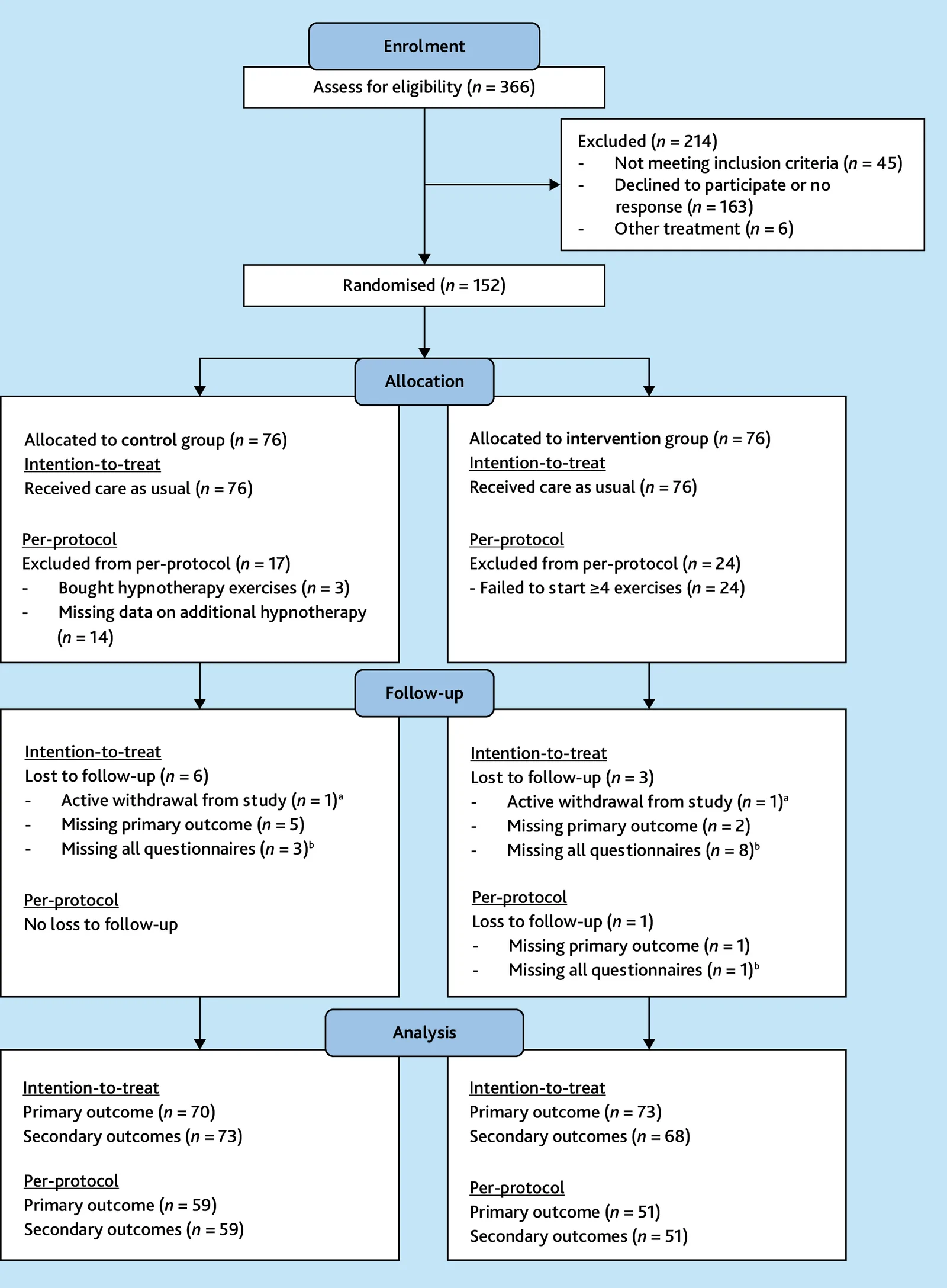
Flowchart of participants. ^a^Data from participants to date of withdrawal were used for secondary outcome analyses, but primary outcome data were missing. ^b^Children with missing follow-up data for all timepoints were not included in analyses for secondary outcomes. Bought hypnotherapy exercises = number of children who had independently purchased and downloaded hypnotherapy exercises from an external website.

In total, 366 children with abdominal pain were informed about the study: 89 presented to their GP and 277 were recruited via (social) media. Of these, 152 children were included for randomisation: 46 recruited by GPs and 106 recruited via media, evenly distributed between groups (Figure 1). The median age of children was 9.4 years (interquartile range 8.2–11.3) and 97 were female (63.8%).

PP analyses excluded 17 children (22.4%) from the control group and 25 children (32.9%) from the intervention group (Figure 1). Baseline characteristics are shown in Table 1. Missing data were present across outcomes, with 5.9% (*n* = 9/152) missingness for the primary outcome. Data from abdominal pain diaries were particularly limited, despite mean imputation, resulting in a reduced sample (*n* = 72/152, 47.4%; *n* = 78/152, 51.3% and *n* = 63/152, 41.4% missing at 3, 6, and 12 months, respectively). No adverse events were spontaneously reported.

**Table 1.** Baseline characteristics of study participants

Baseline characteristic	Valid *N*	Control group	Valid *N*	Intervention group
**Age (years), median (IQR)**	76	9.5 (8.3–11.3)	76	9.1 (8.1–11.2)
**Female**	76	46 (60.5)	76	51 (67.1)
**Rome diagnosis^a^**	76		76	
FAP		29 (38.2)		31 (40.8)
IBS		35 (46.1)		38 (50.0)
AM or FD		10 (13.2)		6 (7.9)
Not fulfilling Rome criteria		2 (2.6)		1 (1.3)
**School level**	76		76	
Primary education		63 (82.9)		62 (81.6)
Secondary education		12 (15.8)		14 (18.4)
Vocational education		1 (1.3)		—
**Pain duration (years), median (IQR)**	70	2.6 (1.4–4.7)	72	2.1 (0.7–4.5)
**History at paediatrician^b^**	69	19 (27.5)	72	17 (23.6)
**School absence**	76	38 (50.0)	76	43 (56.6)
**Pain severity, median (IQR)**	76	6.0 (4.0–7.0)	76	5.0 (4.0–7.0)
**Abdominal pain, median (IQR)**				
Pain intensity score	67	12.0 (7.0–14.0)	73	10.0 (7.0–15.0)
Pain frequency score	67	11.0 (6.0–16.0)	73	9.0 (7.0–17.0)
**Health-related quality of life, median (IQR)**				
Physical wellbeing	76	47.1 (41.1–55.9)	76	47.1 (41.1–54.9)
Psychological wellbeing	76	48.9 (42.0–58.2)	76	48.9 (43.3–57.4)
Moods and emotions	76	41.5 (34.6–48.6)	76	43.9 (36.2–51.3)
Self-perception	76	52.3 (44.7–61.1)	76	49.1 (44.2–61.1)
Autonomy	76	51.0 (47.2–57.1)	76	51.0 (45.7–57.1)
Parent relations and home life	76	52.1 (47.0–58.5)	76	52.1 (46.9–58.5)
Social support and peers	76	53.0 (46.4–60.4)	76	50.7 (46.4–55.4)
School environment	76	52.1 (43.8–57.0)	76	52.1 (43.8–62.5)
Social acceptance (bullying)	76	50.6 (38.3–58.8)	76	44.8 (39.3–58.8)
Financial resources	76	62.9 (49.9–65.0)	76	59.3 (51.9–65.0)
**Pain beliefs, median (IQR)**				
Pain threat	75	2.2 (1.7–2.6)	76	2.2 (1.5–2.7)
Problem-focused coping potential	75	1.5 (0.8–2.0)	76	1.3 (0.7–2.0)
Emotion-focused coping potential	75	2.3 (1.8–3.0)	76	2.3 (1.8–3.0)
**Anxiety score, median (IQR)**	75	12.0 (8.0–18.0)	76	11.0 (7.0–17.0)
**Depression score, median (IQR)**	75	3.0 (2.0–4.0)	76	3.0 (1.0–5.0)
**Somatisation score, median (IQR)**	75	13.0 (7.0–24.0)	76	13.5 (7.5–20.8)
**Sleep self-report**				
Q1: Do you fall asleep in about 20 minutes?	75		76	
Usually (5–7)/week		26 (34.7)		24 (31.6)
Sometimes (2–4)/week		24 (32.0)		27 (35.5)
Rarely (0–1)/week or never		25 (33.3)		25 (32.9)
Q2: Do you wake up at night when your parents think you are asleep?	75		76	
Rarely (0–1)/week or never		47 (62.7)		48 (63.2)
Sometimes (2–4)/week		16 (21.3)		17 (22.4)
Usually (5–7)/week		12 (16.0)		11 (14.5)
Q3: Do you feel sleepy during the day?	75		76	
Rarely (0–1)/week or never		38 (50.7)		38 (50.0)
Sometimes (2–4)/week		28 (37.3)		28 (36.8)
Usually (5–7)/week		9 (12.0)		10 (13.2)
**Treatment expectations, median (IQR)^c^**				
Treatment effect NRS-11, child	72	7.0 (6.0–8.0)	74	7.0 (5.0–8.0)
Treatment effect NRS-11, parent 1	76	7.0 (6.0–8.0)	75	7.0 (6.0–8.0)
Treatment effect NRS-11, parent 2	71	7.0 (6.0–8.0)	69	7.0 (5.0–8.0)
**Treatment preference, child**	73		75	
Preference for intervention		52 (71.2)		52 (69.3)
Preference for control		1 (1.4)		2 (2.7)
No preference		20 (27.4)		21 (28.0)
**Treatment preference, parents**	152		152	
Preference for intervention		118 (77.6)		113 (74.3)
Preference for control		1 (0.7)		2 (1.3)
No preference		27 (17.8)		31 (20.4)
Missing		6 (3.9)		6 (3.9)

Values are *n* (%) unless otherwise indicated. ^a^Three children did not fulfil Rome criteria (reported 2–3 days of abdominal pain in past 2 months, required ≥4). ^b^Prior paediatric consultations were permitted, but ongoing specialist care at baseline was an exclusion criterion. ^c^Range treatment expectations: 0–10. AM = abdominal migraine. FAP = functional abdominal pain. FD = functional dyspepsia. IBS = irritable bowel syndrome. IQR = interquartile range. NRS = numerical rating scale.

**Table 2.** Outcomes for abdominal pain in the intention-to-treat population

Outcome	Valid *N*	Control group	Valid *N*	Intervention group	Crude OR/Beta^a,b^ (90% CI)	Adjusted OR/Beta^a,b^ (90% CI)
**Adequate relief, *n* (%)**						
3 months	74	42 (56.8)	71	52 (73.2)	2.75 (1.24 to 6.10)^c^	2.74 (1.22 to 6.13)^c^
6 months	70	41 (58.6)	67	52 (77.6)	3.28 (1.42 to 7.58)^c^	4.26 (1.81 to 10.04)^c^
12 months	70	52 (74.3)	73	58 (79.5)	1.51 (0.64 to 3.53)	1.73 (0.73 to 4.13)
**Pain severity, median (IQR)**						
3 months	68	5.0 (2.0–6.0)	62	4.0 (1.8–6.0)	−0.36 (−1.07 to 0.35)	−0.31 (−1.00 to 0.38)
6 months	63	5.0 (3.0–6.0)	53	3.0 (1.5–5.0)	−1.40 (−2.14 to −0.65)^c^	−1.38 (−2.11 to −0.66)^c^
12 months	62	5.0 (2.0–6.0)	67	3.0 (1.0–5.0)	−0.91 (−1.62 to −0.20)^c^	−0.89 (−1.57 to −0.20)^c^
**Pain intensity score, median (IQR)**						
3 months	41	5.8 (2.0–10.8)	39	5.0 (1.0–9.3)	−0.62 (−2.57 to 1.34)	−1.06 (−2.89 to 0.77)
6 months	41	4.0 (0.0–10.0)	33	4.0 (0.5–10.9)	−0.50 (−2.51 to 1.51)	−1.18 (−3.07 to 0.70)
12 months	46	5.0 (2.0–8.0)	43	2.0 (0.0–7.0)	−1.58 (−3.47 to 0.31)	−2.19 (−3.96 to −0.42)^c^
**Pain frequency score, median (IQR)**						
3 months	41	5.6 (1.5–11.8)	39	3.5 (0.1–10.0)	−0.90 (−3.11 to 1.30)	−1.55 (−3.53 to 0.43)
6 months	41	3.3 (0.0–10.2)	33	5.0 (0.5–9.7)	−0.13 (−2.39 to 2.13)	−1.13 (−3.17 to 0.90)
12 months	46	4.8 (1.3–9.3)	43	2.0 (0.0–7.0)	−1.73 (−3.86 to 0.40)	−2.82 (−4.73 to −0.91)^c^

^a^Adequate relief – odds ratio; pain severity, intensity and frequency – beta. ^b^Adjusted for age, baseline pain severity score, child’s expectations, and corresponding baseline score. ^c^Significant at alpha of 0.1. CI = confidence interval. IQR = interquartile range. OR = odds ratio.

In the intervention group, 58 of 73 children (79.5%) reported adequate relief after 12 months compared with 52 of 70 children (74.3%) in the control group, with an aOR of 1.47 (90% CI = 0.74 to 2.93) in the ITT population (Supplementary Table S1). Results for the complete cases analysis and pooled estimates on multiple imputed data in both the ITT and the PP populations were similar (Supplementary Table S1).

The intervention group showed more favourable scores on measures related to abdominal pain, including adequate relief, pain severity, frequency, and intensity at almost all time points, although not all differences were statistically significant (Table 2). For example, the proportion of children with adequate relief was higher in the intervention group compared with the control group at 3 months (aOR 2.74, 90% CI = 1.22 to 6.13) and 6 months (aOR 4.26, 90% CI = 1.81 to 10.04). Abdominal pain severity scores were lower in the intervention group at 3 months (aBeta −0.31, 90% CI = –1.00 to 0.38), 6 months (aBeta –1.38, 90% CI = –2.11 to –0.66), and 12 months (aBeta –0.89, 90% CI = –1.57 to –0.20). Similarly, pain threat was lower in the intervention group than in the control group, and more children in the intervention group usually fell asleep within 20 min than in the control group at 3, 6, and 12 months (Supplementary Table S2). Scores on the other secondary outcomes were generally similar between groups (Supplementary Table S2). Similar results were observed when the analyses were repeated in the PP population (Supplementary Table S3).

A post hoc sensitivity analysis with 114 children in the younger age group showed similar results (Supplementary Table S4). No differences were found between boys and girls in the post hoc subgroup analysis by gender (Supplementary Table S5). In the post hoc subgroup analysis by recruitment, the aOR in the GP-recruited children was higher compared with the aOR in media-recruited children, but confidence intervals were broad and showed overlap (aOR 3.10, 90% CI = 0.69 to 13.80 versus aOR 1.19, 90% CI = 0.53 to 2.67, respectively; Supplementary Table S6).

## Discussion

### Summary

In both study groups, a high percentage of children (≥74%) reported adequate relief after 12 months. However, only children in the intervention group had high proportions of adequate relief as early as 3 and 6 months. In addition, these children reported lower abdominal pain scores, including severity, intensity, and frequency during follow-up compared with the control group, reported lower pain threat, and more often reported to usually falling asleep within 20 min.

### Strengths and limitations

To the authors’ knowledge, this was the first trial evaluating the effectiveness of hypnotherapy in children with FAP or IBS in primary care. Its pragmatic design is a strength, providing results that are highly relevant to everyday clinical practice. In addition, missingness was low for the primary outcome, because the authors actively followed up on missing questionnaires and the primary outcome. Results from the multiple imputation analysis were consistent with the complete case analysis, suggesting that missing data did not affect the study findings.

One limitation was the smaller-than-intended sample size because of recruitment challenges, which reduced statistical power. To maintain adequate power, the alpha level was increased to 0.1, resulting in a higher risk of false positives and the use of 90% confidence intervals. Given the low-risk nature of hypnotherapy, this was considered acceptable. However, the results should be interpreted with caution, particularly as only secondary outcomes were significant and the adjusted alpha level may have reduced the precision of the findings.

Recruitment via (social) media yielded participants similar to those recruited via GPs, although longer symptom duration and a higher proportion of FAP might have led to a higher OR in GP-recruited children.^15^ Finally, participants, GPs, and researchers were not blinded to treatment allocation, a common issue in psychological research. Bias was minimised by blinding researchers during analysis. However, most children and parents preferred the intervention arm, which makes placebo effects more likely in that group. As the aim was to evaluate the real-world impact, blinding was not relevant, pragmatic trials compare interventions in patients aware of their care, and placebo effects are part of routine care.^24,25^ Such designs are typically undertaken when prior evidence of efficacy exists, which was the case here.

### Comparison with existing literature

Adequate relief after 12 months was reported by a high proportion of children in the intervention group, consistent with a hospital-based study.^13^ Surprisingly, rates of adequate relief in the control group were higher than expected, as previous research showed that 50% of children in primary care still report abdominal pain having an impact on daily activities after 12 months.^6^ Differences in study design and outcome measures may explain this finding: an RCT design was used to assess adequate relief in the current study whereas the previous study used a cohort design to assess the impact on daily functioning.^6^ Although adequate relief and functioning in daily life do correlate, they are different constructs.^6,26,27^ Another possible explanation is the advancements in primary care management. Since the study by Lisman-van Leeuwen *et al* (2013), a new guideline in Dutch primary care may have improved usual care, contributing to higher relief rates in the current study’s control group.^6^

The current findings suggest that hypnotherapy may result in greater pain reductions, lowered pain threat, and faster sleep onset compared with care as usual. Better sleep was also confirmed by children and parents in interviews evaluating their experience of hypnotherapy.^28^ A key advantage of eHealth is that patients can access it anytime and anywhere, enabling children to practise and apply the exercises without guidance.^29,30^ This may help them develop coping strategies and regain a sense of control over symptoms, contributing to earlier relief, sustained reductions in pain over time, and a lessened perception of pain as a threat.

The study findings are consistent with those of previous small trials of hypnotherapy in secondary care, which reported lower pain scores in the intervention group compared with the usual care or active control groups.^9,31,32^ In line with previous research, the current study found similar improvements between groups in school absenteeism and quality of life over time.^9^ Somatisation, sleep, anxiety, and depression were not previously studied in this context to the authors’ knowledge.^9^ The current study findings suggest that these measures may improve over time, although no differences were observed between the intervention and control groups.

### Implications for practice

The study findings suggest that GPs could offer home-based guided hypnotherapy alongside care as usual to help children achieve faster relief via a low-cost, accessible treatment. Many children declined participation, possibly because of unfamiliarity with hypnotherapy or a preference for conventional care, highlighting the need for clear information and shared decision-making to discuss preferences, expectations, and motivations. Within a trusting GP–patient relationship, discussing the option of online or therapist-delivered hypnotherapy can help tailor care.^33,34^ Further research should identify which children benefit most, including diagnosis and potential cultural influences, and should explore possible adverse effects through qualitative work to inform structured monitoring, complemented by larger-scale observational or registry-based studies to detect rarer adverse events. The authors’ accompanying economic evaluation showed the intervention to be cost-effective, supporting its relevance for implementation in primary care (details available from the authors on request).

## Data

De-identified participant data and a data dictionary will be available upon reasonable request to the corresponding author (g.a.holtman@umcg.nl), from the date of publication and for at least 5 years thereafter. The study protocol and statistical analysis plan will also be shared upon request. Access is subject to review by the study team and a steering committee, based on scientific quality, relevance, and consistency with participant consent. Data will be shared under a data access agreement.The dataset is registered in the UMCG Research Register and has been assigned a DOI via DataCite: https://doi.org/10.34760/6836d2cdaa04e.

## References

[1] Korterink JJ, Diederen K, Benninga MA, Tabbers MM (2015). Epidemiology of pediatric functional abdominal pain disorders: a meta-analysis. PLOS ONE.

[2] Spee LAA, Lisman-Van Leeuwen Y, Benninga MA (2013). Prevalence, characteristics, and management of childhood functional abdominal pain in general practice. Scand J Prim Health Care.

[3] Campo JV, Bridge J, Ehmann M (2004). Recurrent abdominal pain, anxiety, and depression in primary care. Pediatrics.

[4] Scharff L (1997). Recurrent abdominal pain in children: a review of psychological factors and treatment. Clin Psychol Rev.

[5] Albeda FB, Burgers JS, De Jonge AH (2012). Buikpijn bij kinderen [Abdominal pain in children]. https://richtlijnen.nhg.org/standaarden/buikpijn-bij-kinderen.

[6] Lisman-van Leeuwen Y, Spee LAA, Benninga MA (2013). Prognosis of abdominal pain in children in primary care — a prospective cohort study. Ann Fam Med.

[7] Gieteling MJ, Bierma-Zeinstra SMA, Passchier J, Berger MY (2008). Prognosis of chronic or recurrent abdominal pain in children. J Pediatr Gastroenterol Nutr.

[8] Ansems SM, Berger MY, Pieterse E (2023). Management of children with non-acute abdominal pain and diarrhea in Dutch primary care: a retrospective cohort study based on a routine primary care database (AHON). Scand J Prim Health Care.

[9] Abbott RA, Martin AE, Newlove-Delgado TV (2017). Psychosocial interventions for recurrent abdominal pain in childhood. Cochrane Database Syst Rev.

[10] Groen J, Gordon M, Chogle A (2025). ESPGHAN/NASPGHAN guidelines for treatment of irritable bowel syndrome and functional abdominal pain — not otherwise specified in children aged 4-18 years. J Pediatr Gastroenterol Nutr.

[11] Gordon M, Sinopoulou V, Tabbers M (2022). Psychosocial interventions for the treatment of functional abdominal pain disorders in children: a systematic review and meta-analysis. JAMA Pediatr.

[12] Elkins GR, Barabasz AF, Council JR, Spiegel D (2015). Advancing research and practice: the revised APA division 30 definition of hypnosis. Int J Clin Exp Hypn.

[13] Rutten J, Vlieger AM, Frankenhuis C (2017). Home-based hypnotherapy self-exercises vs individual hypnotherapy with a therapist for treatment of pediatric irritable bowel syndrome, functional abdominal pain, or functional abdominal pain syndrome: a randomized clinical trial. JAMA Pediatr.

[14] Wilson S, Delaney BC, Roalfe A (2000). Randomised controlled trials in primary care: case study. BMJ.

[15] Ganzevoort IN, Berger MY, Benninga MA (2025). Using media to enhance paediatric patient recruitment for research in primary care. Prim Health Care Res Dev.

[16] Ganzevoort IN, Fokkema T, Mol-Alma HJ (2023). Home-based guided hypnotherapy for children with functional abdominal pain and irritable bowel syndrome in primary care: study protocol for a randomised controlled trial. BMJ Open.

[17] Schulz KF, Altman DG, Moher D, CONSORT Group (2010). CONSORT 2010 statement: updated guidelines for reporting parallel group randomised trials. BMJ.

[18] Zwarenstein M, Treweek S, Gagnier JJ (2008). Improving the reporting of pragmatic trials: an extension of the CONSORT statement. BMJ.

[19] Hyams JS, Di Lorenzo C, Saps M (2016). Childhood functional gastrointestinal disorders: child/adolescent. Gastroenterology.

[20] Gonsalkorale WM (2006). Gut-directed hypnotherapy: the Manchester approach for treatment of irritable bowel syndrome. Int J Clin Exp Hypn.

[21] van Tilburg MAL, Chitkara DK, Palsson OS (2009). Audio-recorded guided imagery treatment reduces functional abdominal pain in children: a pilot study. Pediatrics.

[22] Rubin DB (1987). Multiple imputation for nonresponse in surveys.

[23] Twisk J, de Boer M, de Vente W, Heymans M (2013). Multiple imputation of missing values was not necessary before performing a longitudinal mixed-model analysis. J Clin Epidemiol.

[24] Monaghan TF, Agudelo CW, Rahman SN (2021). Blinding in clinical trials: seeing the big picture. Medicina (Kaunas).

[25] Sterne JAC, Savović J, Page MJ (2019). RoB 2: a revised tool for assessing risk of bias in randomised trials. BMJ.

[26] Mangel AW, Hahn BA, Heath AT (1998). Adequate relief as an endpoint in clinical trials in irritable bowel syndrome. J Int Med Res.

[27] Saps M, van Tilburg MAL, Lavigne JV (2016). Recommendations for pharmacological clinical trials in children with irritable bowel syndrome: the Rome foundation pediatric subcommittee on clinical trials. Neurogastroenterol Motil.

[28] Ganzevoort IN, van der Veen AL, Alma MA (2025). Children’s and their parents’ experiences with home-based guided hypnotherapy: qualitative study. JMIR Pediatr Parent.

[29] Fisher E, Law E, Dudeney J (2019). Psychological therapies (remotely delivered) for the management of chronic and recurrent pain in children and adolescents. Cochrane Database Syst Rev.

[30] Richardson PA, Harrison LE, Heathcote LC (2020). MHealth for pediatric chronic pain: state of the art and future directions. Expert Rev Neurother.

[31] Vlieger AM, Menko-Frankenhuis C, Wolfkamp SCS (2007). Hypnotherapy for children with functional abdominal pain or irritable bowel syndrome: a randomized controlled trial. Gastroenterology.

[32] Weydert JA, Shapiro DE, Acra SA (2006). Evaluation of guided imagery as treatment for recurrent abdominal pain in children: a randomized controlled trial. BMC Pediatr.

[33] Boland L, Graham ID, Légaré F (2019). Barriers and facilitators of pediatric shared decision-making: a systematic review. Implement Sci.

[34] Ansems SM, Ganzevoort IN, van Tol DG (2023). Qualitative study evaluating the expectations and experiences of Dutch parents of children with chronic gastrointestinal symptoms visiting their general practitioner. BMJ Open.

